# Approach to the Dynamics of the Vocal Cords During the Exploration With Flexible Laryngeal Stroboscopy

**DOI:** 10.1002/lio2.70219

**Published:** 2025-09-09

**Authors:** Walter Tenesaca, Roberto Fernández‐Baillo, Isabel Cardoso, Vicente Pino, Emerson Chachi

**Affiliations:** ^1^ Department of Otorhinolaryngology and Head and Neck Surgery Vithas Arturo Soria Hospital Madrid Spain; ^2^ Department of Otorhinolaryngology and Head and Neck Surgery Campo Arañuelo Hospital Cáceres Spain; ^3^ Department of Surgery, Anatomy and Medical Sciences, Faculty of Medicine and Surgery, Chair of Human Anatomy and Embryology University of Alcalá Madrid Spain

**Keywords:** biomechanical analysis of the voice, free edges, laryngeal stroboscopy, mucosal wave, vocal cords

## Abstract

**Objective:**

The mucosal wave (MW) is fundamental to assessing vocal fold function, with laryngeal stroboscopy (LS) serving as a pivotal tool in its evaluation. However, technical factors associated with LS may alter the basal oscillatory dynamics of the free edges (FEs), which may bias the interpretation of results. This study aimed to analyze the dynamics of FEs, particularly MW, from the correlates extracted using a voice signal analysis method during LS, comparing it with habitual phonation without LS. Additionally, it was determined if this method can complement LS.

**Methods:**

This descriptive study analyzed voice recordings from a sample of 137 adult subjects categorized into three groups with different conditions in their vocal folds. Voice recordings were processed using the Voice OnlineLab VCS tool. All participants had their speech signal recorded at natural pitch and volume in two situations: phonation without LS and phonation during LS. We studied the parameters: F0, tension index, glottal gap size, and MW index, closed/open phases. Statistical analysis was conducted based on the distribution values and mean differences according to the phonation situation, group, and gender. Pearson's correlation coefficient was employed to establish relationships between parameters, and Student's *t*‐test was used to identify differences and test hypotheses.

**Results:**

During LS, mean F0 values increased significantly. In addition, variations were observed in the correlates that reflected the degree of tension, contact type, and the resulting MW. Furthermore, distinct compensatory patterns were identified between male and female participants.

**Conclusions:**

The findings indicate important changes in patients' phonatory gestures during LS. These alterations may bias clinicians' assessments of FEs oscillatory dynamics. Therefore, practitioners using LS in clinical practice should consider these findings.

**Level of Evidence:**

Level 2.

## 1 Introduction

Understanding the mucosal wave (MW) is essential when studying voice disorders [1, 2, 3, 4]. Imaging findings are crucial for visualizing the anatomy and the dynamics of the free edges (FEs) of the vocal folds (VFs). However, imaging alone is insufficient; tools that complement the study of MW are also required [5]. This is fundamental because of the direct association between the MW and the biomechanical properties of the FEs, established by the body‐cover model and determined by their molecular and histological structure [6, 7], being indicators of the stress and flexibility of the underlying tissue; it is essential to produce a high‐quality voice [8]. Currently, several methods are available to study the MW [1, 8]: imaging studies are clearly the gold standard, with laryngeal stroboscopy (LS) being the most widely used tool. In recent years, advanced technologies such as video‐chemography [1, 9, 10] and high‐speed imaging have also emerged [11, 12, 13]. Despite these advancements, LS remains the primary tool owing to its cost‐effectiveness and efficiency, offering real‐time insights into FEs dynamics and the characteristics of the vowel cycle [5, 8, 14, 15, 16, 17, 18]. LS is still considered an effective tool for the differential diagnosis of most voice disorders [11, 13]. However, even though LS is indispensable, it does present certain limitations. First, the image obtained is a visual reconstruction of the vocal cycle from different capture points taken at different consecutive cycles. The representation of this reconstructed cycle depends on the patient's ability to maintain a periodic and stable vibratory pattern during the scan [3, 8, 12, 19]. Second, regardless of the technique used (rigid or flexible), endoscopy involves introducing an invasive element into the phonatory tract. This procedure is not equally well tolerated by all patients, as individual tolerance levels and any anatomical challenges must be taken into account [20, 21, 22]. In most patients, the voice produced during the examination—and consequently, the resulting FEs dynamics—may not reveal an adequate representation of the patient's usual phonation mode [20, 23]. One of the most commonly observed changes is the elevation of the larynx, which significantly alters glottal behavior, a critical factor for achieving effective closure during phonation [21]. Consequently, changes in FEs contact, glottic tension, or subglottic pressure control are observed [24, 25], likely resulting in a modified MW as well. Finally, these direct imaging methods have limitations in quantifying the observed MW [5, 8, 18], since the results often depend on the subjectivity and experience of the observing clinician, introducing a potential bias. To correct this bias, different scales have recently emerged to provide common evaluation criteria. Examples include the VASQ MW score [26], the VALI form [27], and the Rosen form [18].

An alternative approach to studying the MW involves methods that indirectly establish the dynamics of the FEs [20, 28]. These methods are based on the analysis of the biological signal of the voice, from the modeling of the FEs grounded in myoelastic theory [28, 29, 30, 31]. Among the different models available [32, 33, 34, 35], the most advanced is the 3‐mass model, which is applied in the biomechanical analysis of the voice (BAV) tool [28, 32]. This model describes the dynamics of each FE as two masses representing the dynamics of the cover, connected by elastic elements (springs), with a damping system attached to a mass representing the body (vocal muscle) [6, 7, 35]. Through this mathematical modeling and with a signal filtering, the glottal source signal can be obtained, allowing inferences from parameters that provide information regarding many characteristics of glottal dynamics [30, 36, 37, 38, 39, 40, 41, 42, 43, 44, 45, 46]. Indirect methods offer the advantage of representing more natural modes of voice production. These methods capture the radiated signal at the lips using a recording device, avoiding the need to introduce invasive elements into the phonatory tract [46]. However, their greatest limitation lies in their inability to visualize FE anatomy or detect potential injuries.

This study aimed to analyze the dynamic characteristics of the FEs, particularly of the MW, using speech signal processing via BAV for evaluating the presence of modifications during phonation with LS compared to habitual phonation without LS. Additionally, it seeks to determine if BAV can complement LS.

## 2 Methods

### 2.1 Study Design and Participants

This cross‐sectional descriptive study was conducted on a sample of patients who attended the outpatient clinic of a tertiary‐level hospital between October 2021 and January 2023. The project was approved by a hospital ethics committee, and informed consent was obtained from all participants. The study included 137 participants aged 18–60 (mean age: 39.8) years. Participants were divided into three groups based on the following inclusion criteria:

- No voice disorder: The patient did not report dysphonia, scored below 10 on the VHI‐10 [47, 48], the LS study revealed no FE lesion, and the VASQ (Vertical Axis, Anteroposterior Axis, Symmetry and Quantity) MW score was between 5 and 6 (normal range) [26];
- Functional disorder: The patient reported a history of dysphonia, scored above 10 in the VHI, imaging studies did not reveal evident FE lesions, and the VASQ MW score values were either within or outside the normal range.
- Organic disorder: The imaging study revealed an FE lesion, regardless of the VHI‐10 score or VASQ MW score values.

Table 1 shows the laryngeal conditions of the sample and the distribution of the study groups.

**TABLE 1:** Distribution of values of the laryngeal characteristics of the subjects and groups included in the study.

Laryngeal condition	*N*	%	Average VASQ MW score
Acute inflammatory	35	25.5	4.71
Nopathology condition	28	20.4	5.5
Pharyngolaryngeal reflux	27	19.7	5.07
Vocal nodules	15	10.9	3.6
Dysphonia with hyperfunction	9	6.5	5.1
Reinke's oedema	9	6.5	3.44
Psychogenic dysphonia	4	3	5
Spasmodic dysphonia	3	2.2	5.3
Post‐surgical reviews	3	2.2	4.33
Leucoplakia	2	1.5	5
Unilateral laryngeal paralysis	1	0.7	6
Intrachordal cysts	1	0.7	4
Total	137	100	

Study groups			
	*N*	%	Average VASQ MW score
No FEs disorders	28	20.4	5.5
Organic disorder	93	67.9	4.52
Functional disorder	16	11.7	5.19
Total	137	100	

*Note:* The mean values of the VASQ MW scores obtained during laryngeal stroboscopy are included. Among the inflammatory cases, disorders related to acute respiratory tract infections were included. Post‐surgical revisions included cases of lesions resected through laryngeal microsurgery, such as polyps, vocal nodules, and Reinke's oedema.

### 2.2 Instruments and Procedures

All participants underwent the following procedures: (a) a voice history, (b) VHI‐10 questionnaire [47, 48], (c) LS applying the VASQ MW score [26], and (d) voice recording in two circumstances (without LS and during LS).

For voice recordings, patients had to remain seated with their heads in a neutral and comfortable position. Recordings were conducted using the OnlineLab VCS tool [45, 46], installed on an Android smartphone device, with a BOYA BY‐M unidirectional microphone placed 15 cm in front of the patient's lips. Background noise levels were kept below 20 dB using a calibrated sound level meter. Under these conditions, patients were instructed to phonate the phoneme for 4 s at natural pitch and volume (first recording).

For VFs imaging, a flexible endoscope video tower with tip chip (Olympus Visera Elite OTV‐S190), connected to a stroboscopic light source (Strobolux III OPTOMIC), was used. Patients were seated in a natural, comfortable head position that allowed full visualization of the entire length of the VFs. Once the image was stabilized, patients were instructed to perform other phonations (second recording) with the same protocol and conditions established for the first recording.

The biomechanical correlates of the FEs from the first and second voice recordings were extracted using the Voice Clinical Systems signal processing tool, also called “BA,” provided in its Type R3 report (Full Biomechanical Report). The BAV parameters included were: F0 (fundamental frequency), tension index, gap size, and MW correlate parameters (closed/open MW index). All these parameters are shown in Table 2.

**TABLE 2:** Study parameters obtained from the full BAV R3 report.

BAV parameters	Represented correlates
Fundamental frequency F0 (Pr1)	Number of times the vocal folds vibrate per second in the analyzed sample. [Hz]
Tension index (Pr8)	Tension associated with glottal closure is dependent on free edge tension. [ur]
Gap size (Pr12)	Incomplete glottal closure in relation to the closed phase (altered closed phase proportion). Correlates with the affected proportion of the glottal space. [ur]
Closed mucosal wave index (Pr17)	Mucosal wave correlation observed during the closing phase. [ur]
Mucosal wave index in opening (Pr18)	Mucosal wave correlation observed during the opening phase. [ur]

*Note:* Biodynamic parameters provided by the Online Lab App correlate with the laryngeal phonatory function [45].

BAV is included in the list of smartphone applications validated for clinical practice [46]. It serves as a valuable tool for screening, diagnosis, and follow‐up activities as part of the medical or rehabilitative treatment of patients with voice disorders. It features the Voice Online Lab VSC App. Using BAV, a set of parameters can be obtained from the processing of a single voice sample. These parameters provide information on fundamental frequency, vocal cycle phases, glottal flow, glottal force and tension, symmetry, mucous wave (MW), and mass effect, among others. The app has a database of normophonic individuals, allowing voice recordings to be compared according to the age and sex of the participants. The BAV requires that the voice signal radiated at the lips corresponds to the/a/phoneme. This sound is selected because it is the most open in all languages and because it leaves the vocal tract in the most constant and open position possible. In this way, the signal coming from the glottal source will suffer fewer modifications when passing through the supraglottic resonator tract, making the obtained signal more representative. Due to the variations in the position of the tongue and lips that have a greater influence on the emitted glottal signal, voice samples obtained from other phonemes are not recommended for the BAV, nor are speech fragments valid. A validated recording protocol exists [45]. The app automatically processes voice samples and requires an internet connection. This tool offers different types of task‐oriented reports: R1 (screening), R2 (rehabilitative treatment follow‐up), and R3 (full biomechanical report) [30, 36, 37, 38, 39, 40, 41, 42, 43, 44, 45].

Distribution values and mean differences were analyzed according to phonation conditions, group, and gender. To establish relationships, Pearson's correlation coefficient was calculated between the BAV parameters studied in both phonation situations. To identify significant differences and test the study hypotheses, the Student's *t*‐test for independent samples was used. A priori, a statistical significance level of *p* < 0.05 was set. Finally, the statistical analysis was conducted using the SPSS version 29 software.

## 3 Results

### 3.1 Changes in F0

An increase in F0 values was observed during LS compared to habitual phonation without LS (*p* < 0.05). This change was independent of gender, affecting males and females equally. These data are shown in Table 3. In the group analysis (Table 4), the increase in mean F0 values was observed in all study groups. However, significant differences were only found in men in the organic disorder group.

**TABLE 3:** Average values of Pr1, Pr8, Pr12, Pr17, and Pr18 according to the phonation condition.

Parameter	Phonation situation	Women				Men			
		Normal interval in women	Average	Standard deviation	*p*	Normal interval in men	Average	Standard deviation	*p*
Fundamental frequency F0 (Pr1)	PWS	180–240	201.11	35.34	0.029	105–139	118.43	17.42	0.006
	PDS		214.86	41.47			127.81	19.64	
Tension index (Pr8)	PWS	1.0–26	83.36	164.2	0.002	1.49–13	99.37	167.82	0.007
	PDS		18.97	70.36			35.89	69.49	
Gap size (Pr12)	PWS	0–1	27.54	29.8	0.01	0–1	12.2	17.38	< 0.001
	PDS		40.29	36.04			30.37	22.75	
Closed MW index (Pr17)	PWS	130–330	129.17	99.44	0.13	170–520	273.32	150.13	0.002
	PDS		106.71	82.16			200.12	102.72	
Opening MW index (Pr18)	PWS	20–65	83.9	57.67	0.033	15–89	104.46	72.55	< 0.001
	PDS		65.94	44.33			53.79	38.43	

Abbreviations: PDS, phonation during stroboscopy; PWS, phonation without stroboscopy.

**TABLE 4:** Mean values of parameters provided by the BAV across different phonation conditions and study groups.

Group	Parameter	Situation	Women				Men			
			Normal interval in women	Average	SD	*p*	Normal interval in men	Average	SD	*p*
Functional disorder group	Fundamental frequency (Pr1)	PWS	180–240	229.875	31.1541	0.372	105–139	128.175	32.2520	0.431
		PDS		244.100	57.0325			131.775	25.5156	
	Tension index (Pr8)	PWS	1.0–26	53.238	97.1729	0.038	1.49–13	190.075	264.6145	0.057
		PDS		4.725	10.9485			26.188	53.0734	
	Gap size (Pr12)	PWS	0–1	38.500	44.9735	0.333	0–1	7.313	10.3597	0.322
		PDS		29.425	36.2657			25.263	19.5083	
	Closed MW index (Pr17)	PWS	130–330	56.6000	48.655	0.685	170–520	331.9625	189.9187	0.011
		PDS		74.5750	60.147			199.8000	80.5854	
	Open MW index (Pr18)	PWS	20–65	102.713	54.3408	0.825	15–89	103.313	60.1999	0.02
		PDS		81.088	39.3586			36.925	90,456	
Organic disorder group	Fundamental frequency (Pr1)	PWS	180–240	196.411	35.4280	0.341	105–139	118.205	14.5268	0.029
		PDS		212.441	40.1235			129.454	20.0842	
	Tension index (Pr8)	PWS	1.0–26	84.061	161.5477	0.014	1.49–13	98.576	172.8020	0.390
		PDS		24.130	81.3528			45.165	80.5655	
	Gap size (Pr12)	PWS	0–1	26.236	28.1292	0.075	0–1	13.000	18.9423	0.810
		PDS		42.280	36.0339			28.603	21.4724	
	Closed MW Index (Pr17)	PWS	130–330	135.986	100.3934	0.169	170–520	260.257	148.4904	0.141
		PDS		106.898	81.1170			203.114	109.3170	
	Open MW index (Pr18)	PWS	20–65	84.820	62.1584	0.199	15–89	109.819	84.2939	0.003
		PDS		66.230	47.8423			56.243	42.4505	
No disorder group	Fundamental frequency (Pr1)	PWS	180–240	203.858	30.0087	0.848	105–139	114.075	12.2773	0.281
		PDS		206.675	29.6971			122.031	14.8427	
	Tension index (Pr8)	PWS	1.0–26	100.183	215.3409	0.017	1.49–13	55.844	43.5837	0.594
		PDS		4.358	8.1863			19.306	43.1875	
	Gap size (Pr12)	PWS	0–1	26.292	26.7502	0.175	0–1	12.781	16.8139	0.340
		PDS		38.258	37.6653			37.006	26.7846	
	Closed MW index (Pr17)	PWS	130–330	145.767	105.6382	0.922	170–520	274.225	134.7601	0.380
		PDS		127.308	98.0496			193.363	102.1330	
	Open MW index (Pr18)	PWS	20–65	30.2801	0.284	0.405	15–89	92.650	45.7130	0.938
		PDS		25.7225	0.284			56.550	37.0242	

Abbreviations: PDS, phonation during stroboscopy; PWS, phonation without stroboscopy.

### 3.2 Changes in Tension and Glottic Contact

Overall, during LS, decreased tension values and worsening of the contact between the FEs (increased glottic gap size) were observed. All these values reached statistically significant differences and were independent of gender (Table 3). In the group analysis (Table 4), changes in tension reached statistically significant differences in all female study groups and in men in the functional disorder group.

**FIGURE 1:**
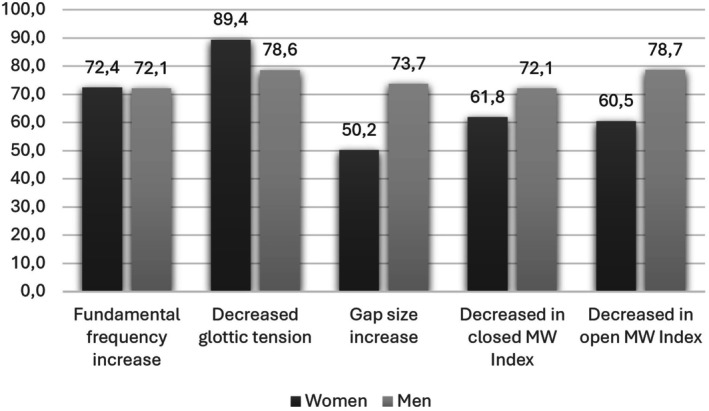
Bar graphic showing the percentage distribution of the population with changes in fundamental frequency, glottal tension, glottal gap, and MW during LS phonation.

Figure 1 shows the changes identified during LS. Changes in F0 had a similar frequency in both genders. However, changes in the glottic gap were found more frequently in the male population, while tension changes were observed more frequently in females.

### 3.3 Changes in MW

**FIGURE 2:**
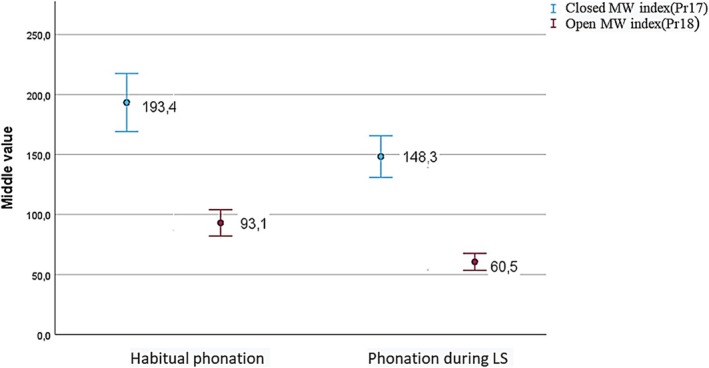
Simple error bar graph (95% CI) illustrating the mean values of Pr17 and Pr18 according to the different phonation conditions in the study population.

All values (closed/opening MW index) decreased during phonation with LS compared to habitual phonation without LS (Figure 2), with statistically significant differences. These differences were more pronounced in the male population compared to the female population (*p* < 0.05), affecting both phases of the vocal cycle. In females, differences were also observed in both phases, but statistical significance was found only in the open phase. All these results are shown in Table 3.

Changes in MW‐related parameters during LS showed statistically significant differences in men in the organic disorder group (open MW index) and also in men in the functional disorder group (open and closed MW indexes; Table 4).

The MW visualized by the examiner during LS yielded different VASQ MW score values for each study group and gender. As shown in Table 1, in the group without FEs disorders and the functional disorder group, the mean MW values were within the normal range (between 5 and 6 VASQ MW score), whereas in the organic disorder group, the observer perceived decreased MW scores (< 5 VASQ MW score).

### 3.4 Relationship Between F0, Glottic Closure Tension, Glottic Gap, and MW

Tables 5 and 6 show the correlation of the biodynamic parameters studied in both genders under the different phonation conditions, in habitual phonation without LS, and phonation during LS, respectively. Considering the reference ranges of Pearson's correlation coefficient and with values reaching statistical significance, the following relationships were found:

- During phonation without LS, MW in the closed phase showed a moderate negative correlation with F0 and a moderate positive correlation with glottal closure tension. During LS, these correlations decreased in females (*p* < 0.05) and males (*p* > 0.05). Glottal gap size showed a moderate negative correlation with MW in the closed phase and was present in both phonation situations and genders. These correlation levels increased significantly during LS.
- A strong negative correlation was observed between MW in closed and open phases in both phonation conditions in the study population, reflecting their inverse proportionality and complementary nature. During LS, MW in the open phase showed a moderate positive correlation with the size of the glottal gap in the whole population.
- During LS, the relationship between glottic gap size and the tension increased in men (moderate negative correlation), whereas in women, this correlation decreased to a very low negative correlation.
- In men, F0 showed a low correlation with glottal closure tension in both phonation conditions. A low correlation was also observed between F0 and glottal gap size during LS.

**TABLE 5:** Correlation table. Values of the correlation coefficients of Pr1, Pr8, Pr12, Pr17, and Pr18 in habitual phonation without LS in women (*N* = 76) and men (*N* = 61).

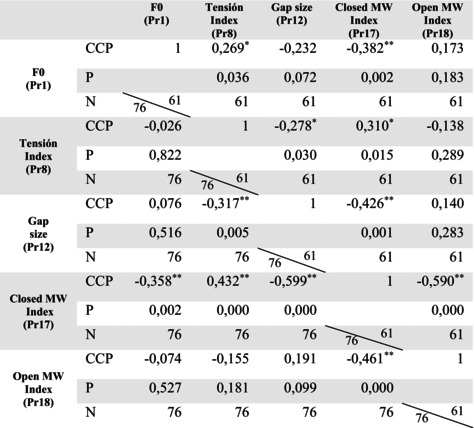

**TABLE 6:** Correlation table. Values of the correlation coefficients of Pr1, Pr8, Pr12, Pr17, and Pr18 in phonation during LS in women (*N* = 76) and men (*N* = 61).

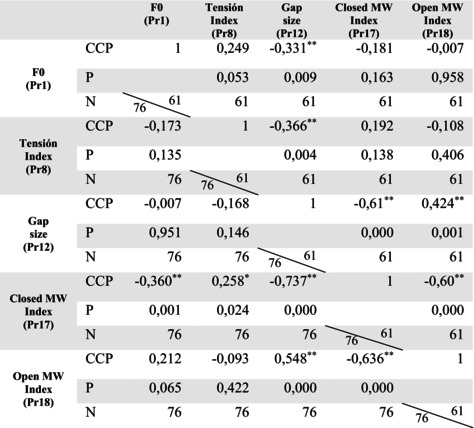

## 4 Discussion

It is well‐known that there are differences in several parameters in the normophonic population, including F0, amplitude, glottic gap [20, 21, 25], anteroposterior laryngeal constriction [20], symmetry [8, 49], as well as in the MW, when analyzed by age [50], gender [50, 51], professional voice use [49, 52, 53]; differences have also been reported when comparing different direct and indirect imaging methods [8, 12, 20, 21, 25, 49].

Understanding the normal limits of the vibratory characteristics of the VFs is important. Equally important is recognizing the variability triggered by certain conditions, such as the use of endoscopic techniques during LS. There is limited scientific evidence about this theme. The results of applying BAV modeling in both phonatory conditions revealed significant variations in the dynamic behavior of the FEs during LS.

Regarding the increase in F0 observed during LS compared to the F0 under baseline conditions of the study population, when analyzing each study group separately, F0 values increased across all groups, with significant differences observed in men with organic FE disorders. This increase in F0 can be explained by the passive elongation of the FEs caused by the action of the cricothyroid (CT) muscle, as described later. Another reason is the elevation of the larynx resulting from the head and neck posture adopted by the patient during LS [54, 55]. It is known that structural alterations of the FEs affect their dynamic properties, as well as F0, glottic gap, and MW [32, 56, 57, 58, 59].

The analysis of factors correlated with changes in F0 during the vowel cycle, tension, and glottal gap [56] revealed significant modifications during LS. The evaluation of the tension showed decreased mean values during LS. This decrease was more evident in females, regardless of their study group.

Regarding EFs contact (glottal gap), several studies have reported the presence of a physiological glottal gap [20, 21, 25]. There are also studies describing that the glottal gap correlates with decreased loudness and elevated fundamental frequency [60, 61]. Our findings showed a significant worsening of the glottal gap during LS. While these results were consistent across genders, the effect was more pronounced in the male population. This may have a considerable impact, as glottal gaps observed during LS are more likely to be exaggerated than the physiological ones, especially in males. In addition, the results revealed the correlations between tension and the glottal gap, and also between the glottal gap and the MW. During LS, when the active tension of the FEs decreases, the size of the glottal gap increases, and the MW decreases. As previously detailed, BAV data allow us to make inferences about the dynamic behavior of the FEs. In this study, the parameters provided by the BAV show biomechanics characterized by an increase in fundamental frequency, a decrease in the MW, a decrease in FEs tension, and an increase in the glottic gap during LS compared to normal phonation without LS.

This biomechanical behavior fits with the type II phonatory model or register, which is characterized by the longitudinal stretching of the FEs carried out by the unopposed action of the cricothyroid muscle, which leads to an increase in the fundamental frequency [62, 63]. The action of the CT and thyroarytenoid (TA) muscles in the adjustment of phonatory modes is well known [4, 57, 58, 64]. The vocal behavior pattern during LS, as demonstrated with the aid of BAV in this study's population, bears a degree of similarity to the phonatory mode or register that occurs during falsetto, where elongation and partial separation of the FEs occur without opposition from the vocalis muscle, which increases the frequency [32, 65, 66]. Further research is needed to further investigate this topic.

The most clinically relevant findings emerged when analyzing the MW correlate. During phonation with LS, MW values decreased in both phases, and this reduction was more pronounced in men, especially in those with an organic or functional disorder of FEs. MW decreases during high‐pitch phonation [57, 64, 67]. Elongation of the VF reduces cover flexibility, and increased oscillation velocity decreases the time for the cover to vibrate [4, 68, 69, 70, 71]. In addition, VFs are longer and thicker in males than in females, leading to baseline differences in MW [44]. Structural or dynamic alterations in the FEs reduce the cover's vibration capacity.

A correlation was evident between the MW with F0, tension, and glottic gap, observing a balance between them, which becomes altered during LS. During LS, MW in the closed phase decreases owing to the increase in F0, the decrease in active tension of the FEs, and fundamentally owing to the increase in the glottic gap.

Taking into account the VASQ MW score, the observer perceived normal MW values in patients without FE alterations and in those with functional FE disorders, whereas decreased MW values were observed primarily in patients with organic FE disorders. In addition to the LS information, the BAV provided important information by differentiating the open/closed phases of the MW and offering insights into F0, tension, and glottic gap dynamics. Therefore, we believe that BAV could serve as a valuable complement to LS.

Regarding the endoscopic technique, Ng and Bailey [25] studied the acoustic changes produced during LS with a rigid endoscope, demonstrating that F0, percentage of phase fluctuation, and noise‐to‐harmonics ratio were significantly increased. Lim et al. [20] studied the effects of flexible laryngeal endoscopy on vocal performance in young women using acoustic parameters, finding alterations in respiratory control, pitch, and voice quality, including decreased maximum phonation time and increased F0. These investigations concluded that acoustic alterations in the vocal output signal mean that the visual output signal may not be representative. Paulus et al. [21] compared LS using a rigid and flexible endoscope, finding less glottic gap and better visualization of the anterior commissure with the flexible endoscope. Although current imaging techniques attempt to replicate physiological conditions as closely as possible [25], the inherently invasive nature of endoscopic techniques makes it necessary to consider noninvasive tools to assess vocal physiology [20]. It is important to assess the voice using samples produced at or near the habitual pitch of phonation to ensure that the results are representative [25].

The invasive nature of endoscopy, the mechanical stimulation and/or anxiety associated with this procedure, can alter cervical and laryngeal posture, affecting the oscillatory behavior of the FEs, as well as examiner instructions to seek better exposure, prompting the patient to increase volume and pitch [23, 72]. It is also possible that genders respond differently to phonatory gesture compensation during LS. In men, the VFs are less exposed during LS owing to the lower position of the larynx and the sharper thyroid angulation. This may lead to greater upward movement of the larynx to improve visualization [21, 50, 51].

Given the stable histological structure of the VFs during adulthood, this study excluded younger subjects (< 18 years) and older adults (> 60 years) owing to histological variations associated with VF maturation and aging, respectively [73].

Several studies highlight the growing interest in combining imaging techniques with other noninvasive methods. This would improve the approach to patients with voice disorders [1, 8, 20, 24, 51, 74, 75].

## 5 Conclusions

The results confirm that in patients undergoing LS, F0, tension, FE contact, and the MW are altered compared to habitual phonation, with distinct compensatory patterns observed between males and females. These findings should be considered to avoid misjudgment in cases where the MW phenomenon is critical for diagnosis and therapeutic planning. BAV is a tool that provides valuable data and could be highly useful in complementing LS. Therefore, these results may serve as a basis and a justification for future studies.

## Ethics Statement

The study protocol was reviewed and approved by the Ethical Committee for Research with Medicines of HM Hospitales Madrid, Spain (CEIm HM Hospitales) under code 23.12.2265‐GHM. We confirm that all research was conducted in accordance with relevant guidelines and regulations.

## Consent

Written informed consent was obtained from all participants.

## Data Availability

All data generated or analyzed in this study are the responsibility of the authors. To request the data, further inquiries can be directed to the corresponding author.
